# Irisin Suppresses Nicotine-Mediated Atherosclerosis by Attenuating Endothelial Cell Migration, Proliferation, Cell Cycle Arrest, and Cell Senescence

**DOI:** 10.3389/fcvm.2022.851603

**Published:** 2022-04-08

**Authors:** Junye Chen, Kang Li, Jiang Shao, Zhichao Lai, Ran Gao, Chaonan Wang, Xitao Song, Wenjun Guo, Xiaoxi Yu, Fenghe Du, Zhan Zhu, Jiaxian Wang, Jiangyu Ma, Leyin Xu, Yan Zhou, Jianghao Liu, Keqiang Shu, Hongmei Zhao, Jing Wang, Bao Liu

**Affiliations:** ^1^Department of Vascular Surgery, Peking Union Medical College Hospital, Peking Union Medical College, Chinese Academy of Medical Sciences, Beijing, China; ^2^State Key Laboratory of Medical Molecular Biology, Institute of Basic Medical Sciences, Chinese Academy of Medical Sciences, Department of Pathophysiology, Peking Union Medical College, Beijing, China; ^3^Eight-Year Program of Clinical Medicine, Peking Union Medical College Hospital, Peking Union Medical College, Chinese Academy of Medical Sciences, Beijing, China; ^4^Four-Year Program of Clinical Medicine, Peking Union Medical College Hospital, Peking Union Medical College, Chinese Academy of Medical Sciences, Beijing, China

**Keywords:** nicotine, atherosclerosis, irisin, senescence, P53

## Abstract

Atherosclerotic disease has become the major cause of death worldwide. Smoking, as a widespread independent risk factor, further strengthens the health burden of atherosclerosis. Irisin is a cytokine that increases after physical activity and shows an atheroprotective effect, while its specific mechanism in the process of atherosclerosis is little known. The reversal effect of irisin on intimal thickening induced by smoking-mediated atherosclerosis was identified in *Apoe*^–/–^ mice through the integrin αVβ5 receptor. Endothelial cells treated with nicotine and irisin were further subjected to RNA-seq for further illustrating the potential mechanism of irisin in atherosclerosis, as well as the wound healing assays, CCK-8 assays, β-gal staining and cell cycle determination to confirm phenotypic alterations. Endothelial differential expressed gene enrichment showed focal adhesion for migration and proliferation, as well as the P53 signaling pathway for cell senescence and cell cycle control. Irisin exerts antagonistic effects on nicotine-mediated migration and proliferation *via* the integrin αVβ5/PI3K pathway. In addition, irisin inhibits nicotine-mediated endothelial senescence and cell cycle arrest in G0/G1 phase *via* P53/P21 pathway. This study further illustrates the molecular mechanism of irisin in atherosclerosis and stresses its potential as an anti-atherosclerotic therapy.

## Introduction

Cardiovascular and cerebrovascular diseases are the main causes of death in elderly individuals worldwide (1). Atherosclerosis (AS) is the main pathological process. A retrospective study reported that in 2020, approximately 28% of the global population aged 30–79 had carotid intima-media thickening (thickness ≥1.0 mm), affecting more than 1 billion people. In addition, approximately 21% of people (∼816 million) have carotid plaque, 1.5% of people (∼58 million) have carotid artery stenosis (2). With urbanization and an aging population, it is estimated that the number of patients with carotid atherosclerosis in China will reach 267 million in 2020, accounting for 30.07% of people aged 30–79, an increase of 70% over that in 2000 (3). It is urgent to develop drugs or methods that can interfere with the progression of atherosclerosis.

Smoking is an independent risk factor for AS disease. As many as 48.7% of people over the age of 15 smoke passively in China, aggravating the harm of tobacco (4). Nicotine in tobacco is involved in the vascular inflammatory response. We previously found that nicotine can promote vascular intima-media thickening *via* the PI3K pathway (5). Smoking can also aggravate existing vascular endothelial injury, and nicotine can induce the loss of normal vascular endothelial function. Nicotine is associated with increased expression and decreased activity of nitric oxide synthase protein in endothelial cells, which leads to excessive production of endogenous reactive oxygen species (ROS) (6, 7). Under nicotine stimulation, ROS in vascular endothelial cells react directly with nitric oxide to form peroxynitrite, resulting in protein nitration and atherosclerosis (6, 8).

Fibronectin type III domain-containing protein 5 (FNDC5) is a transmembrane glycoprotein that can be hydrolyzed and cleaved to endogenously secrete irisin (9). Irisin is closely related to atherosclerosis. In patients with Behcet’s disease, a decrease in irisin levels is a risk factor for carotid intima thickening (10), suggesting that irisin secretion increases in the early stage of chronic inflammatory disease to overcome the inflammatory state of the body and plays a protective role. In hyperglycemia and hypertension models, irisin can stimulate endothelial cells to secrete nitric oxide and play a protective role in vasodilation (11). In a murine atherosclerotic model, irisin reduced autophagy and the infiltration of macrophages and T cells in atherosclerotic plaques, including reduced expression of inflammatory factors in atherosclerotic plaques (12). Irisin regulates the focal adhesion kinase (FAK) signaling pathway [including FAK (*Ptk2*), a downstream protein between integrin receptor and PI3K] through the formation of complexes with CD81, integrin α1β1 and αVβ5 receptors. Loss of CD81 can affect the role of irisin in antagonizing obesity, insulin resistance, and adipose tissue inflammation, thus accelerating atherosclerosis, indicating the atheroprotective effect of irisin through integrin αVβ5 (13). Previously, we found that irisin can antagonize nicotine-induced intima-media thickening and macrophage infiltration during atherosclerosis by inhibiting the activation of PI3K after binding with integrin αVβ5 receptors (5). Irisin has been shown to play a role by binding to the integrin αVβ5 receptor of human umbilical vein endothelial cells (HUVECs) (14). Although irisin has been found to be associated with atherosclerosis in disease phenotype and circulating irisin level has been used to evaluate prognosis in a number of clinical trials, the molecular mechanism by which irisin inhibits atherosclerosis is still unclear.

In this study, we further explored the protective effect and mechanism of irisin on nicotine-mediated atherosclerosis through RNA-seq and *in vivo* and *in vitro* assays. We investigated the effects of irisin on lipid deposition in aortic roots, evaluated its effects on vascular intimal thickness, and explored the key pathway regulated by irisin. Finally, we found that irisin inhibits nicotine-induced atherosclerosis by affecting endothelial migration, proliferation, the cell cycle and senescence.

## Materials and Methods

### Experimental Animals

All animal experiments were performed based on the guidelines of the National Health and Family Planning Commission of the People’s Republic of China and approved by the Animal Ethics Committee of Peking Union Medical College Hospital (approval number JS-2335).

Six-week-old male *Apoe*^–/–^ C57BL/6J mice (The Charles River Laboratories, United States) were randomly divided into five groups: (I) Control group: *n* = 8; (II) Nicotine group: nicotine (No. N3876, Sigma-Aldrich, China) was approached in drinking water at 100 μg/mL for 8 weeks, *n* = 8; (III) Irisin group: irisin (No. 11451, Cayman, United States) was administered at 2 μg in 100 μL normal saline per mouse intravenously (i.v.), twice a week from week 5, *n* = 8; (IV) Nicotine + Irisin group: nicotine and irisin were applied as mentioned above, *n* = 8; and (V) Nicotine + Irisin + Cilengitide group: 4 μg/g cilengitide (HY-16141, MedChemExpress, China) in 100 μL normal saline, intraperitoneally (i.p.), approved every other day from week 5, in addition to nicotine and irisin, *n* = 8. Cilengitide contains an Arg-Gly-Asp (RGD) binding motif, which could specifically bind to integrin αVβ5 and play an inhibitory role (15). The dosage and manner of administration of nicotine, irisin and cilengitide in mice refer to our previous study (5). All of the *Apoe*^–/–^ mice were fed in a standard SPF chamber at 20–26°C, humidity range of 40–70%, 12 h light/dark cycle and were free access to food and water. All mice were fed with high-fat diet (D12492, The Charles River Laboratories, United States) for the later 8 weeks. The groups without nicotine intervention were given saline in the same manner. Serum irisin level was detected by ELISA kit (ZC-55012, ZCIBIO, China) after the euthanasia of mice.

### Oil Red O Staining

En-face Oil red O staining: aortas of two mice from each group were separated and fixed for 24 h. The vessels were cut longitudinally along the vessel wall and stained with Oil Red O liquid for 60 min at 37°C. The vessels were then incubated with 75% ethanol until the atherosclerotic lesions turned red and the vessel wall became white. Cross section Oil red O staining: Aortic roots from six mice embedded in OCT were serially cross sectioned (5 μm thick sections) and stained with Oil Red O (G1261-2, Solarbio, China) to evaluate the lesion size and lipid contents. After circling the outline of blood vessels (including whole valves), Image pro plus (Ver. 6.0, Media Cybernetics, MD, United States) was used to calculate the total area of blood vessels (including positive staining area) based on total area, and then the positive area is calculated based on Oil red O under the same contour, and finally the positive area/total area of blood vessels is calculated. Images were captured by Pannoramic DESK (3DHISTECH, Hungary) and preprocessed by CaseViewer (Ver. 2.4, 3DHISTECH, Hungary) and analyzed by Image-Pro Plus (Ver. 6.0, Media Cybernetics, MD, United States).

### Immunofluorescent Staining

For immunofluorescence, sections from the abdominal aorta (*n* = 6) were incubated with rabbit anti-CD31/PECAM-1 antibody (1:100, NB100-2284, Novus, United States) and then secondary CY3-conjugated goat anti-rabbit IgG (1:300, GB21303, Servicebio, China). The outer layer area and inner layer area of intima were measured by Image pro plus (Ver. 6.0, Media Cybernetics, MD, United States) based on positive CD31 staining, and the intimal thickness was obtained by subtracting the radius of inner layer from the radius of outer layer. Fluorescence was scanned by Pannoramic DESK (3DHISTECH, Hungary). Image preprocessing was conducted by CaseViewer (Ver. 2.4, 3DHISTECH, Hungary).

### Cell Culture and Treatment

Human umbilical vein endothelial cells (National Infrastructure of Cell Line Resource, IMBS, CAMS/PUMC) were cultured in endothelial cell medium (ECM, No. 1001, ScienCell, United States) containing 1% endothelial cell growth supplement (ECGS, No. 1052, ScienCell, United States), 5% fetal bovine serum (FBS, No. 0025, ScienCell, United States) and 1% penicillin/streptomycin solution (No. 0503, ScienCell, United States) in 5% CO_2_ at 37°C. HUVECs were administered in continuous two phases. Phase I, cells were treated with 10^–6^ molL^–1^ nicotine for 24 h, as our previous research (16), in Nicotine group as well as Nicotine + Irisin group, while Control group and Irisin group were administrated with standard ECM. Then in phase II, cells were treated with irisin (20 nM for 24 h), as previously described (17), in Irisin group as well as Nicotine + Irisin group; Nicotine group and Nicotine + Irisin group were treated with nicotine (10^–6^ molL^–1^ for 24 h), while HUVECs in Control group were cultured with equal volume of standard ECM.

### Wound Healing Assay

Human umbilical vein endothelial cells were cultured and treated as mentioned above in 6-well plates and were subjected to wound healing assays. After 24 h culture of Phase I, three straight lines were drawn in each well with 200 μL tips. The cell culture supernatant was discarded, and the cells were gently washed three times with fresh medium. Replacement of corresponding medium 2 mL/well in phase II, 37°C, 5% CO_2_ cell incubator. In phase II, at 0, 6, and 12 h with the same magnification (40×), the cells were observed and photographed under a microscope to monitor cell migration. ImageJ software was used to measure the baseline and wound area at each time point. Each experiment was performed in triplicate.

### CCK-8 Assay

Cell proliferation was detected using a Cell Counting Kit-8 (CK04, Dojindo, Japan) according to the manufacturer’s specifications. Cells were seeded at concentrations of 5 × 10^4^/well in 96-well plates and cultured as mentioned above. After the culture of phase I in each group, supernatants were discarded at 6 and 24 h of phase II with serum-free ECM washing, and CCK-8 working fluid (CCK-8: serum-free ECM = 1:10) was added at 100 μL per well and incubated at 37°C for 2–4 h according to the color change. The OD values (450 nm) were measured by a microplate reader (Multiskan FC, Thermo Fisher, United States). Each experiment was performed in triplicate.

### Cell Cycle Detection

A PI Cell Cycle Detection kit (GK3601, Genview, China) was used to determine the cell cycle state. After interventions, cells were collected and washed with D-PBS once. Cells were resuspended in 300 μL PI staining fluid and incubated for 15 min at 37°C. Washing cell with DPBS once and resuspending cells. Samples were analyzed with a flow cytometer (NoVocyte, ACEA Biosciences Inc., China). Data analysis was conducted by FlowJo (V10.6.2, Becton Dickinson & Company, United States).

### β-Gal Staining

Senescence β-Galactosidase Staining Kit (C0602, Beyotime, China). For cells cultured in a 6-well plate, the cell culture medium was removed and the cells were washed once with PBS. Then, 1 mL galactosidase stain fixative was added, and the cells were incubated at room temperature for 15 min. The cells were washed with PBS for 3 min each time. Then, 1 mL of working fluid was added to each well. Incubation at 37°C overnight without CO_2_. Observation and cell counting were conducted by optical microscopy and ImageJ software (ver. 1.44, National Institutes of Health, United States).

### RNA-Seq

Human umbilical vein endothelial cells cultured with different interventions were dissolved in TRIzol reagent and subjected to RNA sequencing (Novogene, China). RNA integrity was detected by RNA Nano 6000 Assay Kit of the Bioanalyzer 2100 system (Agilent Technologies, United States). Sample sequencing library fragments were produced after synthesis and amplification of cDNA. Finally, PCR products were purified (AMPure XP system, Beckman Coulter, United States), and library quality was controlled (Bioanalyzer 2100 system, Agilent Technologies, United States). Clustering of the index-coded samples was performed on a cBot Cluster Generation System using TruSeq PE Cluster Kit v3-cBot-HS (Illumina) according to the manufacturer’s instructions. Subsequently, the library preparations were sequenced (Illumina NovaSeq) and 150 bp paired-end reads were generated. Quality control and adaptor removal were conducted on Fastq data. The index of the reference genome was built by Hisat2 (v2.0.5), and paired-end clean reads were aligned to the genome (GRCh38). Read numbers mapped to each gene were counted by featureCounts (v1.5.0-p3). Differentially expressed genes (DEGs) among groups were identified by using DESeq2 (1.20.0). *P*-values ≤ 0.05 adjusted by the Benjamini and Hochberg approach and | log2fold change (FC)| ≥ 1 were significant gene expression criteria in this study (18). Volcano plot was visualized by Sangerbox tools^1^ with ggplot 2. Gene Ontology (GO) enrichment analysis and KEGG^2^ enrichment analysis were implemented by the clusterProfiler (3.4.4) R package and KOBAS (19). Terms with corrected *P*-values less than 0.05 were considered significantly enriched by DEGs (20).

### Western Blot

Proteins were lysed and extracted by RIPA buffer (89900, Thermo Scientific, United States). Subsequently, proteins were separated by SDS-PAGE and transferred to PVDF membranes. The primary antibodies used for immunoblotting were PI3K (1:4,000, YM3408, Immunoway, China), AKT (1:4,000, 4691, CST, United States), p-AKT (S473, 1:4,000, YP0006, Immunoway, China), P53 (1:4,000, Ab26, Abcam, China), P21 (1:2,000, ab109199, Abcam, China), P16^*INK*4*a*^ (1:5,000, ab108349, Abcam, China), GAPDH (1:5,000, YM3029, Immunoway, China), and β-Actin (1:5,000, YM3028, Immunoway, China). The immunoreactive bands were visualized *via* electrochemiluminescence (ECL, DW101-01, TransGen Biotech, China) method and analyzed by ImageJ software (ver. 1.44, National Institutes of Health, United States).

### Quantitative RT-PCR

Sample RNA extraction was performed by TRIzol total RNA extraction reagent (15596-018, Invitrogen, United States). The PrimeScript™ RT reagent Kit (TaKaRa, RR036A, Japan) and TB Green Premix Ex Taq II (TaKaRa, RR820A, Japan) were used for cDNA synthesis and real-time amplification according to the manufacturer’s instructions.

The primer sequences for the target genes were as follows: *CDK1*-F: 5′-AAACTACAGGTCAAGTGGTAGCC-3′, *CDK1*-R: 5′-TCCTGCATAAGCACATCCTGA-3′; *CDKN2A*-F: 5′-GAAG GTCCCTCAGACATCC-3′, *CDKN2A*-R: 5′-GTAGGACCTTCG GTGACTG-3′; *TP53*-F: 5′-CAGCACATGACGGAGGTTGT-3′, *TP53*-R: 5′-TCATCCAAATACTCCACACGC-3′; *CDKN1A*-F: 5′-TTAGCAGCGGAACAAGGAG-3′, *CDKN1A*-R: 5′-AGAAA CGGGAACCAGGACA-3′; *18srRNA*-F: 5′-GTAACCCGTTGA ACCCCATT-3′, and *18sRNA*-R: 5′-CCATCCAATCGGTA GTAGCG-3′; *GAPDH*-F: 5′-CACCCACTCCTCCACCTTTGA-3′, and *GAPDH*-R: 5′-TCTCTCTTCCTCTTGTGCTCTTGC-3′. All data were analyzed with the 2^–ΔΔ*CT*^ method and normalized to the expression level of *GAPDH* or *18sRNA*. All values are presented as fold changes relative to the Control group.

### Statistics

GraphPad Prism (ver. 7.0, GraphPad, United States) was used for data statistics. All results are presented as the mean ± standard deviation (SD). Comparisons between two groups were analyzed using the two-tailed Student’s *t*-test, and comparisons between three or more groups were analyzed using one-way ANOVA. Statistical significance was defined as *P < 0.05, ^**^P < 0.01, ^***^P < 0.001, and ^****^P < 0.0001.

## Results

### Irisin Inhibits Nicotine-Induced Atherosclerosis *in vivo*

**FIGURE 1:**
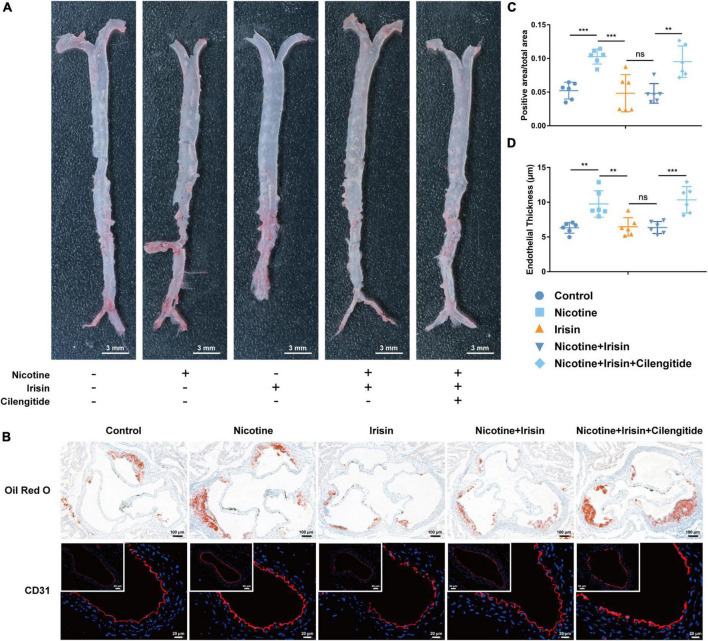
Irisin inhibits nicotine-mediated atherosclerosis in *Apoe–/–* mice. **(A)** En-face Oil red O staining of aortas from the Control group, Nicotine group, Irisin group, Nicotine + Irisin group and Nicotine + Irisin + Cilengitide group, *n* = 2. **(B)** Cross sections of aortic root stained with Oil red O dye and immunofluorescence of CD31. **(C)** Quantification of aortic root with Oil red O staining, *n* = 6. **(D)** Quantification of abdominal aorta stained with CD31, *n* = 6. *P < 0.05, **P < 0.01, ***P < 0.001, and ****P < 0.0001. Data are mean ± SD; ns, no significance.

Forty 6-week-old *Apoe*^–/–^ mice were randomly divided into five groups: the Control group, Nicotine group, Irisin group, Nicotine + Irisin group, and Nicotine + Irisin + Cilengitide group. The mice underwent a high-fat diet and nicotine intervention for 8 weeks, of which the latter 4 weeks were exposed to irisin and/or cilengitide. Murine aortas were harvested to perform en-face Oil red O staining (Figure 1A) and aortic root Oil red O staining (Figure 1B). Comparing Control group, the positive area/total area (including valve + luminal area) of Nicotine group was significantly larger (0.103 ± 0.011 vs. 0.052 ± 0.012, *P* < 0.001, Figure 1C), suggesting the pro-atherosclerotic effect of nicotine. There was no significant difference between Irisin group and Control group (0.048 ± 0.028 vs. 0.052 ± 0.012, *P* > 0.05), indicating that the single use of irisin had no extra effect on atherosclerotic lesion area, while the positive area/total area of Nicotine group was significantly higher than Nicotine + Irisin group (0.103 ± 0.011 vs. 0.048 ± 0.015, *P* < 0.001), suggesting that irisin could reverse nicotine-induced atherosclerosis. As an inhibitor of integrin αVβ5, cilengitide inhibited the effect of irisin, which presented a significantly higher positive area/total area value than Nicotine + Irisin group (0.095 ± 0.023 vs. 0.048 ± 0.015, *P* < 0.01, Figure 1C). Immunofluorescence staining of CD31 (Figure 1B) was performed on cross sections of the abdominal aorta. The results indicated that nicotine could promote intimal thickening (9.751 ± 1.904 vs. 6.313 ± 0.772 μm, *P* < 0.01, Figure 1D), while Irisin group and Nicotine + Irisin group displayed a thinner intima than the Nicotine group (6.465 ± 1.322 vs. 9.751 ± 1.904 μm and 6.357 ± 0.858 vs. 9.751 ± 1.904 μm, *P* < 0.01). Cilengitide attenuated the effect of irisin on the intima (10.35 ± 1.908 vs. 6.357 ± 0.858 μm, *P* < 0.001). Irisin intervention was evaluated by serum irisin levels (Supplementary Figure 1). In general, nicotine promotes the development of atherosclerosis by promoting intimal thickening, whereas irisin inhibits nicotine-mediated intimal thickening and atherosclerosis through integrin αVβ5 receptors.

### Differential Gene Expression and Enrichment Analyses of Endothelial Cells Treated With Irisin

**FIGURE 2:**
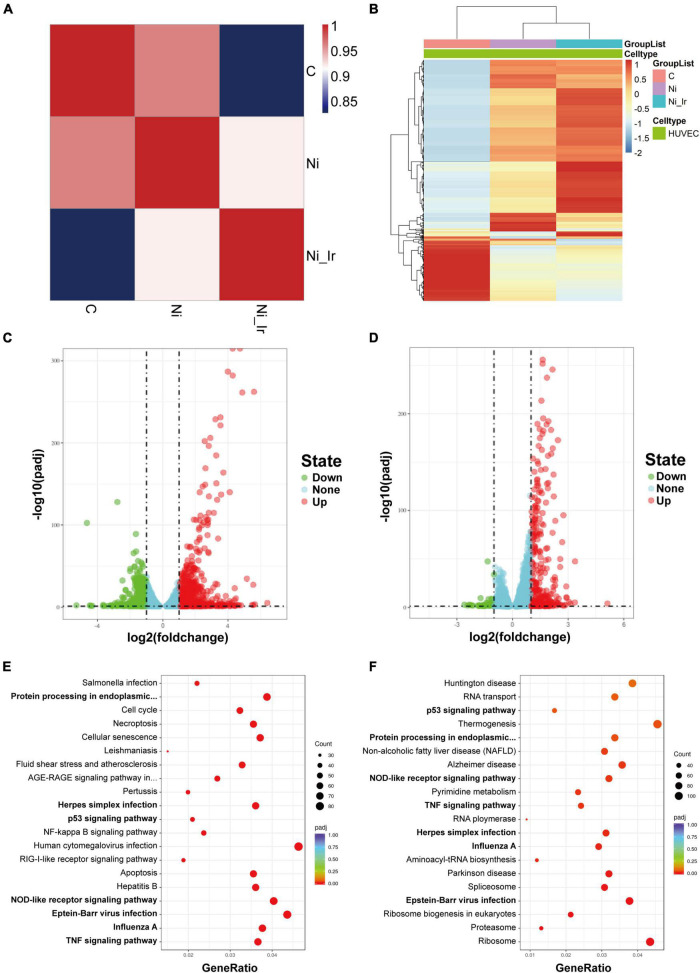
Differentially expressed genes (DEGs) and enrichment analyses of endothelial cells treated with nicotine and irisin. **(A)** Correlation analysis of DEGs in the Control group **(C)**, Nicotine group (Ni) and Nicotine + Irisin group (Ni_Ir). **(B)** Heatmap of the top 1,000 DEGs among the three groups. **(C)** Volcano plot of DEGs between the Ni group and C group (padj < 0.05, | log2fold change| > 1); **(D)** Volcano plot of DEGs between the Ni_Ir group and Ni group (padj < 0.05, | log2fold change| > 1); **(E)** KEGG pathway enrichment analysis of DEGs between the Ni group and C group; **(F)** KEGG pathway enrichment analysis of DEGs between the Ni_Ir group and Ni group.

To study the mechanism of nicotine and irisin on HUVECs, cells were treated with 10^–6^ M nicotine for 24 h, followed by another 24 h of 20 nM irisin (Ni_Ir) or another 24 h of 10^−6^ M nicotine (Ni), and the Control group (C) was cultured in standard medium for the whole 48 h. Cells were collected for RNA-sequencing. To show the global alterations of genes under interventions, three groups of DEGs underwent correlation analysis (Figure 2A) and generated a clustering DEG heatmap (Figure 2B). Compared with the Control group, the Nicotine group had 849 upregulated genes and 451 downregulated genes (padj < 0.05, | log2FC| > 1, Figure 2C). The Nicotine + Irisin group had 324 upregulated genes and 45 downregulated genes compared with the Nicotine group (padj < 0.05, | log2FC| > 1, Figure 2D). KEGG pathway enrichment analysis indicated that the intersection of DEG pathways between the Nicotine group vs. the Control group and the Nicotine + Irisin group vs. the Nicotine group included the TNF signaling pathway (hsa04668), NOD-like receptor signaling pathway (hsa04621), P53 signaling pathway (hsa04115), Influenza A (hsa05164), Epstein-Barr virus infection (hsa05169) and Protein processing in endoplasmic reticulum (hsa04141, Figures 2E,F). The GO database indicated that the intersection of upregulated DEG pathways in the Nicotine group vs. the Control group, and downregulated DEG pathways in the Nicotine + Irisin group vs. the Nicotine group contained signal transduction by P53 class mediators, which belong to the biological processes (BP) category (Supplementary Table 2). The intersection of downregulated DEG pathways in the Nicotine group vs. the Control group and upregulated DEG pathways in the Nicotine + Irisin group vs. the Nicotine group included the regulation of cell cycle arrest and focal adhesion, BP category (Supplementary Table 2). Based on the enriched pathways, we deeply analyzed genes of which expression level reversed in the comparison between Nicotine + Irisin group vs. the Nicotine group and the Nicotine group vs. the Control group. *PIK3C2A* (Ni. vs. C: log2FC = 0.8019, padj = 9.53E-06; Ni_Ir. vs. Ni: log2FC = −0.4577, padj = 4.28E−06), *CDK1* (Ni. vs. C: log2FC = 0.3816, padj = 0.0038; Ni_Ir. vs. Ni: log2FC = −0.5806, padj = 9.46E−15), *CDKN2A* (Ni. vs. C: log2FC = −0.4954, padj = 0.0004; Ni_Ir. vs. Ni: log2FC = 0.2656, padj = 9.43E−05), *CDK4* (Ni. vs. C: log2FC = −0.6596, padj = 7.22E−07; Ni_Ir. vs. Ni: log2FC = 0.2370, padj = 0.0021) and more intersected, in which PAI1 (*SERPINE1*) is a downstream signal of P53. Cyclin E2 (*CCNE2*) forms a complex with CDK2 to regulate the cell cycle. Given that the irisin receptor, integrin αVβ5, is closely related to focal adhesion, as well as the enriched P53 signaling pathway and its downstream gene expression alteration, we hypothesized that irisin may influence endothelial cell proliferation, migration, senescence, and cell cycle control.

### Irisin Suppresses the Migration and Proliferation of Endothelial Cells Induced by Nicotine

**FIGURE 3:**
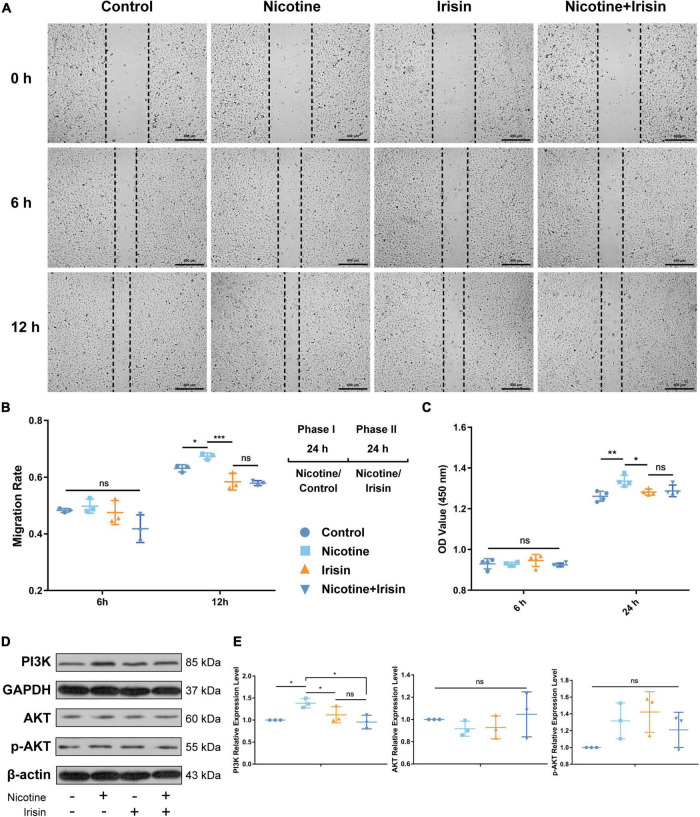
Irisin inhibits nicotine-mediated endothelial cell migration and proliferation. **(A)** Wound healing assay under nicotine, irisin and nicotine + irisin interventions performed at 0, 6 and 12 h during phase II intervention, *n* = 3. **(B)** Migration rate among groups at 6 and 12 h during phase II administration and 2 phases treatment pattern illustration. **(C)** CCK-8 cell proliferation test performed at 6–24 h during phase II intervention, *n* = 4. **(D)** Immunoblotting bands of PI3K, AKT, p-AKT (S473), GAPDH, and β-actin. **(E)** Quantification of PI3K, AKT, and p-AKT. All results were normalized to the expression level of GAPDH or β-actin and presented as the fold change of the Control group, *n* = 3. *P < 0.05, **P < 0.01, ***P < 0.001, and ****P < 0.0001. Data are mean ± SD; ns, no significance.

We found enrichment of focal adhesion, PI3K signaling, cell cycle and other pathways in the RNA-seq results, and we previously confirmed that irisin can antagonize nicotine-induced intima-media thickening and exert its anti-atherosclerotic effect by inhibiting PI3K *via* the integrin αVβ5 receptor in mice (5). In this study, we further confirmed that irisin inhibits nicotine-mediated intimal thickening *via* CD31 immunofluorescent staining. To determine the inhibitory effect of irisin on nicotine-mediated endothelial cell migration and proliferation, wound healing assays (Figure 3A) and CCK-8 tests were carried out at 6, 12, and/or 24 h after intervention. The results showed that 6 h after the beginning of the scratch test, there was no significant difference between the Control group, Nicotine group, and Irisin group (0.483 ± 0.007 vs. 0.498 ± 0.025 vs. 0.475 ± 0.042, *P* > 0.05, Figure 3B). After 12 h of intervention, the migration rate of Nicotine group was higher than the Control group (0.674 ± 0.011 vs. 0.631 ± 0.013, *P* < 0.05), while irisin suppressed the migration rate of the Nicotine + Irisin group compared with that of the Nicotine group (0.579 ± 0.009 vs. 0.674 ± 0.011, *P* < 0.001), which confirmed that irisin could inhibit the migration of endothelial cells mediated by nicotine. After 24 h of intervention, the cell boundaries in each group were enclosed, so they were not evaluated.

The results of the CCK-8 assay (Figure 3C) showed that after 24 h of intervention, Nicotine group presented higher cell viability than the Control group (1.336 ± 0.028 vs. 1.261 ± 0.0245, *P* < 0.01), while irisin reversed the effect of nicotine (1.288 ± 0.029 vs. 1.336 ± 0.028, *P* < 0.05) and maintained no significance with Control group (1.288 ± 0.029 vs. 1.261 ± 0.025, *P* > 0.05). These results indicated that irisin could rescue the proliferation of endothelial cells caused by nicotine in a time-dependent manner.

Western blot analysis (Figure 3D) showed that nicotine upregulated the expression of PI3K in HUVECs (1.382 ± 0.105 vs. 1 ± 0, *P* < 0.05, Figure 3E), while irisin antagonized the upregulation of PI3K induced by nicotine (0.955 ± 0.156 vs. 1.382 ± 0.105, *P* < 0.05). There was no significant difference in the total protein expression of AKT and p-AKT among all groups. It is suggested that irisin inhibits the protein level of PI3K to exert its protective effect on migration and proliferation in endothelial cells mediated by nicotine.

### Irisin Ameliorates Nicotine-Mediated Endothelial Senescence and Cell Cycle Arrest

**FIGURE 4:**
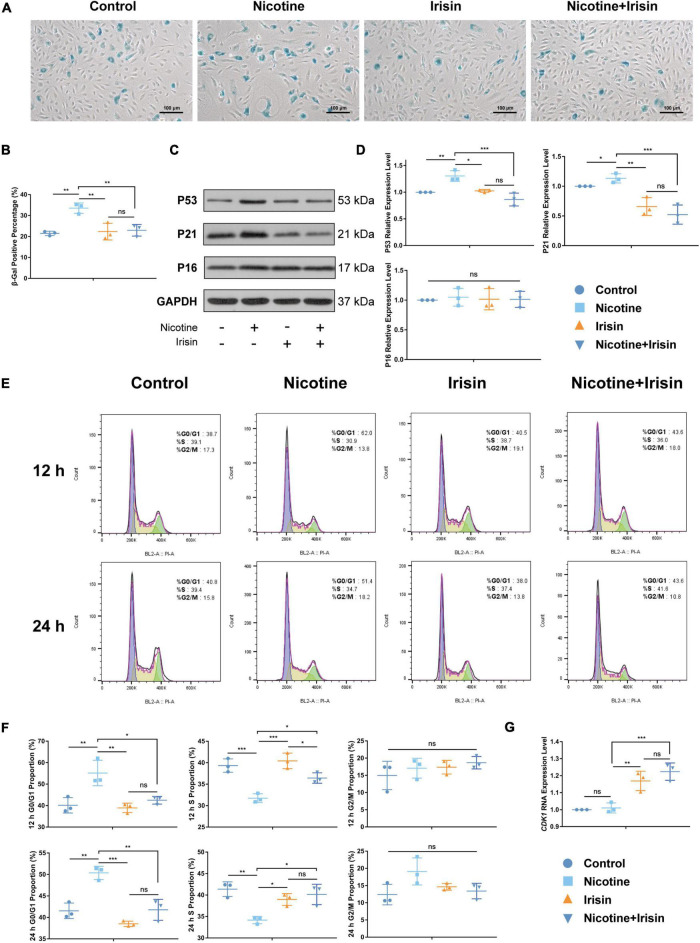
Irisin inhibits nicotine-mediated endothelial cell senescence and cell cycle arrest. **(A)** β-Gal staining of HUVECs after phase I and II interventions. **(B)** Quantification of the β-gal positive staining percentage. **(C)** Immunoblotting bands of P53, P21, P16, and GAPDH after phase I and II interventions. **(D)** Quantification of western blot of P53, P21, and P16. **(E)** Propidium iodide (PI) cell cycle assay performed at 12 and 24 h during phase II intervention. **(F)** G0/G1, S, and G2/M phase proportions at 12 and 24 h during phase II intervention. **(G)** Quantitative RT-PCR of CDK1. All results were normalized to the expression level of GAPDH and presented as the fold change of the Control group. *P < 0.05, **P < 0.01, ***P < 0.001, and ****P < 0.0001. Data are mean ± SD; ns, no significance, *n* = 3.

According to the RNA-seq results, P53 signaling and other pathways were enriched by DEGs. We conjectured that irisin could ameliorate cell senescence and regulate cell cycle to further play an anti-atherosclerotic role. β-Gal staining was conducted to evaluate the aging condition of endothelial cells for three replicants with three photographs randomly captured for each well. The results (Figure 4A) showed that nicotine promoted an increase in the cell senescence phenotype (33.54 ± 2.475 vs. 21.47 ± 1.031%, *P* < 0.01, Figure 4B), while irisin reversed this aging phenotype mediated by nicotine (22.96 ± 2.794 vs. 33.54 ± 2.475%, *P* < 0.05). In terms of mechanism, we detected the protein and mRNA levels of classic markers of cell senescence, including P53 (*TP53*), P21 (*CDKN1A*), and P16 (*CDKN2A*, Figure 4C and Supplementary Figure 2), and found that nicotine could increase the protein expression of P53 (1.305 ± 0.099 vs. 1 ± 0, *P* < 0.01, Figure 4D) and P21 (1.133 ± 0.078 vs. 1 ± 0, *P* < 0.05). Moreover, irisin reduced the protein expression levels of P53 (0.863 ± 0.121 vs. 1.305 ± 0.099, *P* < 0.001) and P21 (0.524 ± 0.161 vs. 1.133 ± 0.078, *P* < 0.001) mediated by nicotine, but there was no significant difference in the expression level of P16 between the three groups at the protein level (1.05 ± 0.150 vs. 1.016 ± 0.179 vs. 1.013 ± 0.135, *P* > 0.05), which was not consistent with the RNA-seq results.

To further clarify the causes of the anti-senescence effect of irisin, we detected the cell cycle with PI staining *via* flow cytometry (Figure 4E). The results showed that nicotine elevated the proportion of G0/G1 phase between the Control group and Nicotine group (55.17 ± 5.92 vs. 40.13 ± 3.573%, *P* < 0.01 for 12 h; 50.37 ± 1.537 vs. 41.53 ± 1.815%, *P* < 0.01 for 24 h, Figure 4F) at 12 and 24 h after intervention. Irisin rescued the G0/G1 proportion mediated by nicotine in a time-dependent manner (42.47 ± 1.793 vs. 55.17 ± 5.92%, *P* < 0.05 for 12 h; 41.77 ± 2.438 vs. 50.37 ± 1.537%, *P* < 0.01 for 24 h). Correspondingly, nicotine intervention decreased the S phase proportion of HUVECs to arrest cell at G0/G1 phase, while irisin abolished the effect of nicotine in a time-dependent manner, since the S phase proportion of Nicotine + Irisin group is lower than Irisin group (36.43 ± 1.21 vs. 40.43 ± 1.804%, *P* < 0.05) at 12 h and it becomes no significance between these two groups (40.13 ± 2.369 vs. 38.97 ± 1.38%, *P* > 0.05) at 24 h after intervention. After irisin intervention, the expression of P21 was downregulated (0.524 ± 0.1611 vs. 1.133 ± 0.078, *P* < 0.001, Figure 4D) compared to that of the Nicotine group, which weakened the ability of cell cycle arrest in G0/G1 phase and promoted the cells to shift from G0/G1 phase to S phase, so the proportion of S phase increased in a time-dependent manner after intervention. The RNA-seq results showed that *CDK1* expression in Nicotine group was higher than that in the Control group (log2FC = 0.3816, padj = 0.0038), while that in the Nicotine + Irisin group was significantly lower than that in the Nicotine group (log2FC = −0.5806, padj = 9.46E−15). CDK1 is known closely associated with G2/M phase passage (21, 22). After verification by RT-PCR, we found the expression in Nicotine + Irisin group was significantly higher than that in Nicotine group (1.224 ± 0.051 vs. 1.01 ± 0.030, *P* < 0.001, Figure 4G), indicating irisin could facilitate cell cycle passage. *CDK4* (Ni. vs. C: log2FC = −0.659569317, padj = 7.22E−07; Ni_Ir. vs. Ni: log2FC = 0.2370, padj = 0.0021) was consistent with the protein level of P21 and supported that irisin rescued nicotine induced G0/G1 phase cell cycle arrest. Overall, the results suggest that nicotine can promote endothelial cell senescence and cell cycle arrest, while irisin can promote cell cycling and inhibit nicotine-mediated senescence through the P53/P21 pathway.

## Discussion

With the growing aging of the population, cardiovascular disease has become the leading cause of death in developing and developed countries (23–26). Smoking is an independent risk factor for atherosclerosis, which causes vascular inflammation, endothelial homeostasis alteration and eventually leads to atherosclerosis (27). Although people’s health awareness and medical level have improved, the prognosis of cardiovascular disease is still not ideal, and there is a lack of effective intervention measures. The level of irisin in blood circulation is related to the progression of vascular atherosclerosis and is considered to be an independent predictor of subclinical atherosclerosis (10, 28–31). However, the specific mechanism of irisin in the process of atherosclerosis is little known. In this study we found that irisin could reverse nicotine-induced lipid deposition, plaque lesion formation, and intimal thickening *in vivo*, while the protective effect of irisin was abolished after the administration of cilengitide, suggesting that irisin acted on endothelial cells through the integrin receptor αVβ5. To further elucidate the anti-atherosclerotic mechanism of irisin on endothelial cells, we performed RNA-seq on HUVECs. Experiments *in vitro* found that irisin could reverse nicotine-mediated endothelial cell migration and proliferation *via* integrin αVβ5/PI3K pathway. Furthermore, it is found that irisin could reduce endothelial senescence-mediated by nicotine through the P53/P21 pathway, which is closely associated with inhibiting G0/G1 phase arrest-mediated by nicotine and promoting cell cycle passage.

**FIGURE 5:**
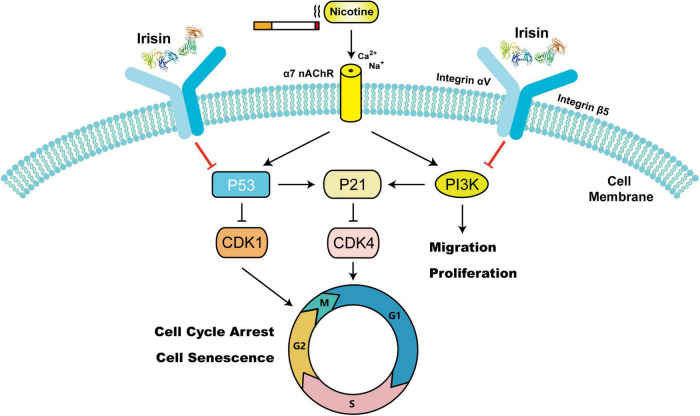
Schematic model of the mechanism by which irisin suppresses nicotine-mediated migration, proliferation, cell cycle arrest, and cell senescence in endothelial cells. [Irisin’s protein data bank (PDB) ID: 4LSD (42, 43)].

This study further verified the way in which irisin enters endothelial cells to play an atheroprotective role. Previously, Kim et al. (32) reported that integrins α1β1 and αVβ5 are potential irisin receptors in osteocytes and adipocytes. Chemical inhibition of αVβ5 integrin receptors can significantly block the signal transduction and function of irisin. In addition, researchers have confirmed that extracellular irisin can be directly absorbed into lung cells from circulation through endocytosis mediated by lipid rafts to prevent lung ischemia–reperfusion injury (33). Our previous studies confirmed that irisin could reverse nicotine-induced aortic intima-media thickening and macrophage infiltration in *Apoe*^–/–^ mice, while the protective effect of irisin was weakened by the administration of cilengitide, an inhibitor of integrin receptor αVβ5 (5). Although 14-week-old *Apoe*^–/–^ mice in this study were only fed a high-fat diet for 8 weeks, which was located at the early stage of atherosclerosis with limited intimal thickening observed, and may lead to the limited reversal effect of cilengitide. The results of aortic root Oil red O staining and abdominal aortic CD31 immunofluorescence staining confirmed that cilengitide could antagonize the atheroprotective effect of irisin and its endothelial cellular entry of integrin αVβ5, which is consistent with previous reports. Nevertheless, there is no study to elucidate the protective mechanism of irisin in smoking or nicotine-related atherosclerosis in endothelial cells. To further elucidate the anti-atherosclerotic mechanism of irisin on endothelial cells, we performed RNA-seq on HUVECs. Proliferation, migration, senescence and cell cycle phenotypes were further investigated. Studies have shown that irisin promotes the proliferation of HUVECs through the ERK signaling (34) and protects endothelial cells from high glucose-induced apoptosis by regulating Bax and Caspase (35) and promoting angiogenesis (9). Irisin regulates the FAK signaling pathway through forming complexes with CD81, integrin α1β1 and αVβ5 receptors, thus promoting the proliferation of mouse beige adipose precursors (13). Interestingly, experiments *in vitro* confirmed that irisin could reverse nicotine-mediated endothelial migration and proliferation rather than promoting proliferation, suggesting that irisin maintains the homeostasis of endothelial cells. Nicotine intervention was found to activate PI3K expression, which serves as a downstream protein of integrin receptors and mainly participates in focal adhesion and regulates cell motility and proliferation (hsa04510, Supplementary Figure 3). There was no significant difference in the total protein level of AKT and p-AKT (S473) between all groups, which did not match the alterations of PI3K and P21, a downstream target of AKT. Considering that the phosphorylation of S473 is related to the complete activation of AKT and that its expression level is similar to the expression pattern of total AKT, irisin may bypass AKT and play a further role in migration and proliferation through other pathways. Given the sequencing results, we detected the cell senescence phenotype of endothelial cells and confirmed that irisin could reverse nicotine-mediated endothelial senescence *via* P53/P21 pathway for the first time. Studies have shown that inhibiting the phenotype of cell aging can delay the progression of atherosclerosis (36, 37). In addition, irisin can inhibit nicotine-mediated cell cycle arrest (G0/G1 phase) and promote endothelial cell cycle shifting in a time-dependent manner, which further confirms that irisin plays an anti-aging role by inhibiting cell cycle arrest and promoting cell cycle passage (Figure 5).

Skeletal muscle is the predominant source of irisin production. The level of serum irisin increases after physical activity and promotes the conversion of white adipose tissue to brown adipose tissue, thus promoting thermogenesis (38). In patients with Behcet’s disease, a decrease in irisin levels is a risk factor for carotid intima thickening (10). Higher irisin serum levels are associated with lower coronary artery calcification (39), coronary heart disease and myocardial infarction (NCT02498431) (40). Together, exercise could increase the level of irisin and delay the aging of blood vessels, which may ultimately affect the whole body at a safe serum concentration. To further clarify the research status of irisin in clinical trials, we searched ClinicalTrials.gov and the Chinese Clinical Trial Registry (ChiCTR) with irisin (Supplementary Table 1). Irisin has not been used as a therapeutic drug for vascular-related diseases. In eight clinical studies related to vascular diseases, the detection of irisin content in serum, plasma or muscle biopsy was used as an evaluation marker. Briefly, in five clinical studies, circulating irisin levels were used to evaluate the prognosis and metabolic levels of cardiovascular diseases such as coronary heart disease and diabetic foot after different treatments. In three other studies, circulating irisin levels were measured in patients with diabetes or renal dialysis to evaluate the relationship between irisin and endothelial dysfunction. The above clinical trials stressed that physical exercise can increase irisin level and play the atheroprotective role. At the same time, the corresponding applications of irisin as a therapy for atherosclerosis require further investigation.

There are several limitations to our study. In order to avoid estrogen affecting the establishment of atherosclerotic animal model (41), only male mice were included in this study, which may not distinguish the potential effects of irisin driven by gender. The interaction between integrin αVβ5 and nicotinic acetylcholine receptor α7 needs to be further studied for a better understanding on the mechanism of irisin antagonizing nicotine-induced atherosclerosis. Irisin suppresses PI3K to inhibit proliferation and migration bypassing AKT, and the specific downstream pathway remains to be further studied for illustrating the ability of endothelial homeostasis maintaining by irisin. The correlation between smoking status and serum irisin levels in clinics needs to be further clarified. The improvement of the above limitations in the future is expected to provide a basis for irisin as a clinical intervention for smoking-related atherosclerosis.

## Conclusion

In summary, we confirmed for the first time that irisin rescues nicotine-mediated endothelial senescence through the P53/P21 pathway, which promotes cell cycle passage and inhibits cycle arrest in G0/G1 phase and ultimately inhibits nicotine-mediated atherosclerosis. Irisin also showed antagonistic effects on nicotine-mediated migration and proliferation of endothelial cells. In addition, irisin has a clear anti-nicotine-mediated atherosclerotic effect in *Apoe*^–/–^ mice *via* integrin αVβ5. Therefore, irisin has the potential to be involved as an anti-atherosclerosis therapy.

## Data Availability Statement

The datasets presented in this study can be found in online repositories. The names of the repository/repositories and accession number(s) can be found below: https://ngdc.cncb.ac.cn/gsa-human/, HRA001771.

## Ethics Statement

The animal study was reviewed and approved by the Ethics Review Committee of Peking Union Medical College Hospital.

## Author Contributions

BL, JW, and HZ supervised the studies, designed the analysis, and revised the final manuscript. JC, KL, CW, JXW, JM, XY, FD, and ZZ performed the experiments *in vitro*. JC, KL, JS, ZL, and XS performed the experiments *in vivo*. JC, RG, WG, JL, LX, and KS performed the data analysis and bioinformatics. JC and KL drafted the manuscript. All authors approved the final manuscript.

## Conflict of Interest

The authors declare that the research was conducted in the absence of any commercial or financial relationships that could be construed as a potential conflict of interest.

## Publisher’s Note

All claims expressed in this article are solely those of the authors and do not necessarily represent those of their affiliated organizations, or those of the publisher, the editors and the reviewers. Any product that may be evaluated in this article, or claim that may be made by its manufacturer, is not guaranteed or endorsed by the publisher.

## Footnotes

1
http://www.sangerbox.com/tool

2
http://www.genome.jp/kegg/
