# Supplementary material for: Irisin Suppresses Nicotine-Mediated Atherosclerosis by Attenuating Endothelial Cell Migration, Proliferation, Cell Cycle Arrest, and Cell Senescence
Source: Front Cardiovasc Med. 2022 Apr 8;9:851603. doi: 10.3389/fcvm.2022.851603 (PMC9023791; doi:10.3389/fcvm.2022.851603)
Supplement: Supplementary file 1 [file Data_Sheet_1.DOCX]

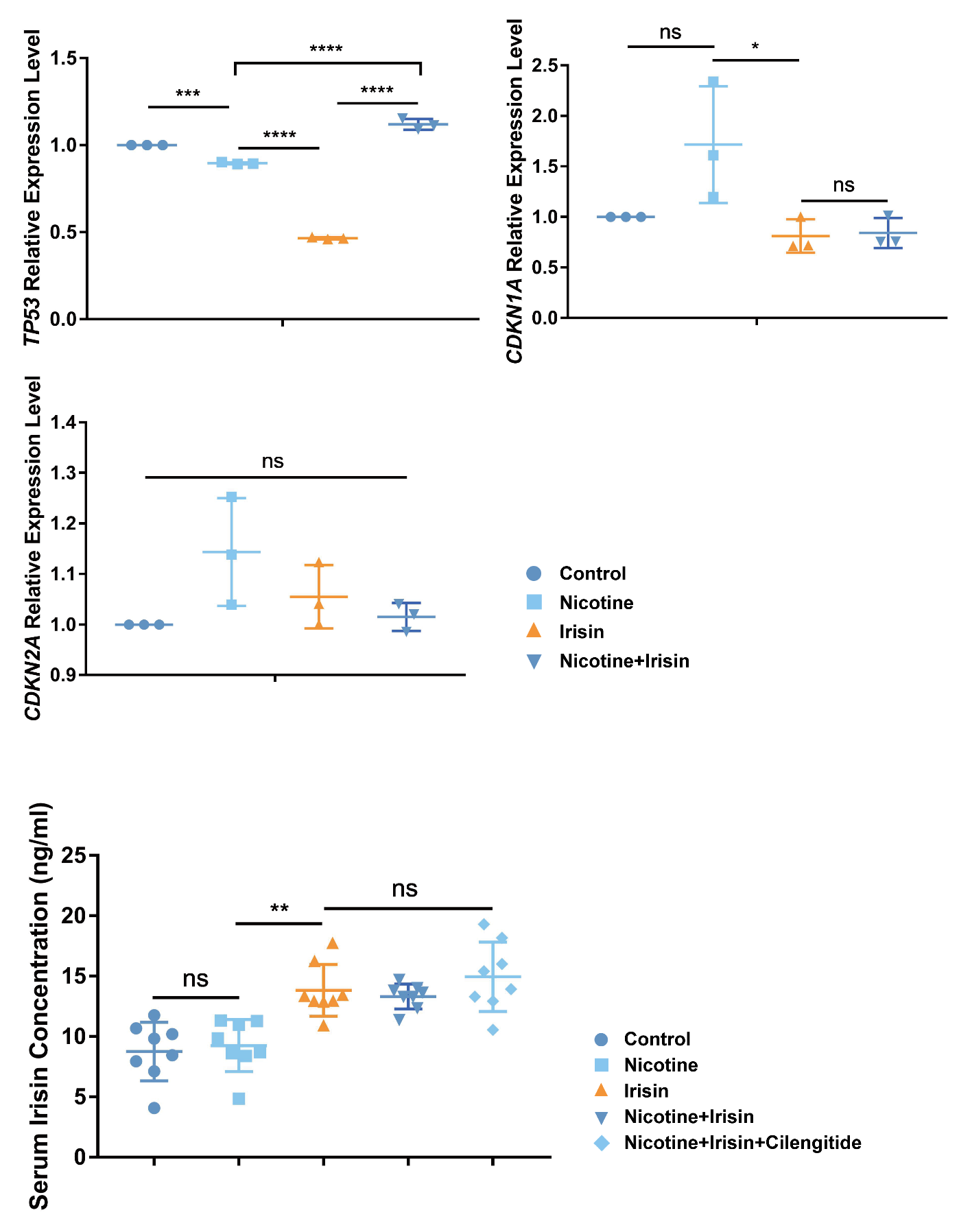


**Supplementary Figure 1. Serum level of irisin in *Apoe-/-* mice.** The quantification of serum irisin level was used to evaluate the irisin intervention. *P<0.05 and **P<0.01 by one-way ANOVA. Data are mean ± SD; ns, no significance.


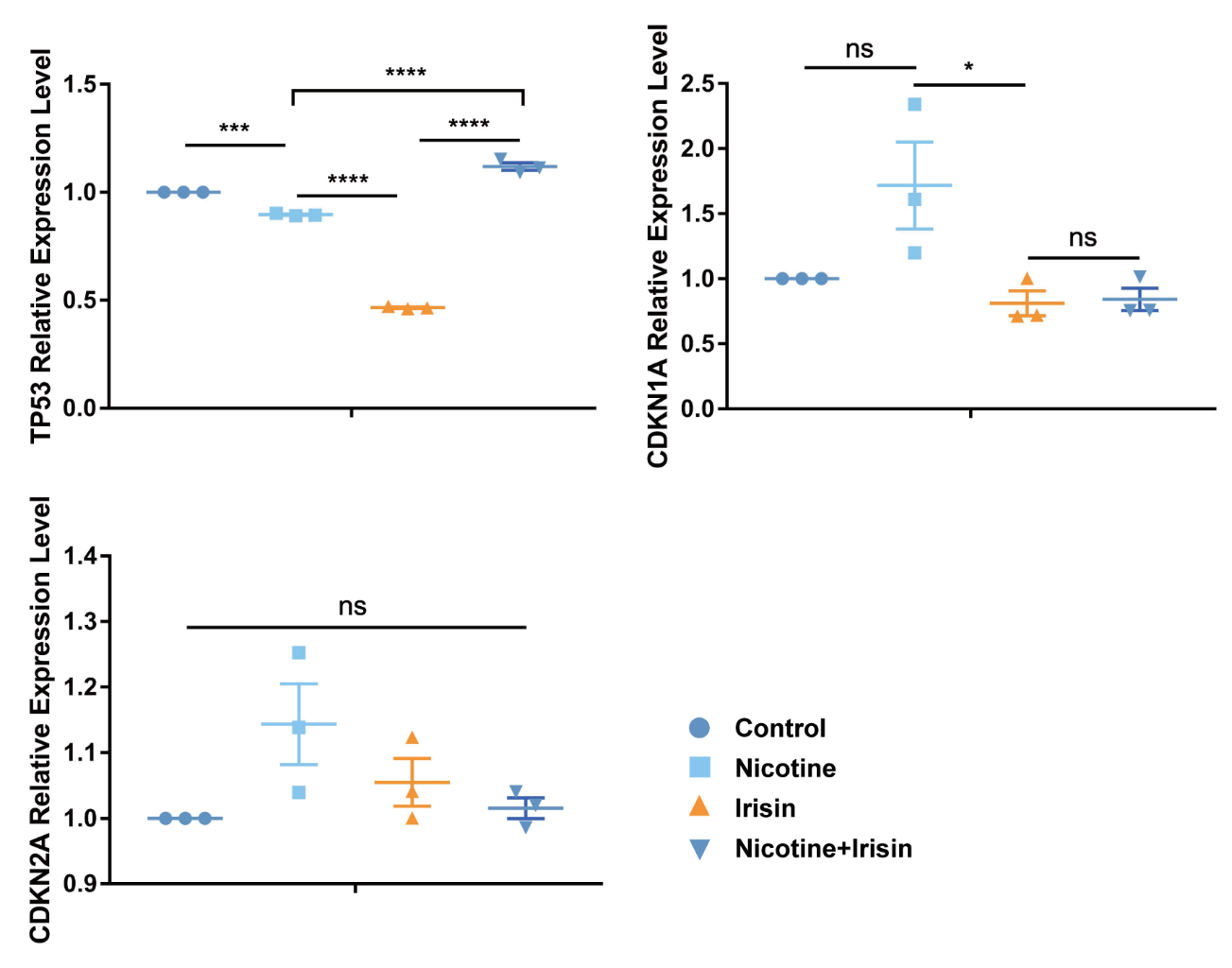


**Supplementary Figure 2. RT-PCR quantification of TP53, CDKN1A and CDKN2A from the Control group, Nicotine group, Irisin group, Nicotine+Irisin group.** All data were analyzed with the 2^-△△CT^ method and normalized to the expression level of GAPDH or 18sRNA. All values are presented as fold changes relative to the Control group. *P < 0.05, **P < 0.01, ***P < 0.001, and ****P < 0.0001. Data are mean ± SEM; ns, no significance, n=3.

**
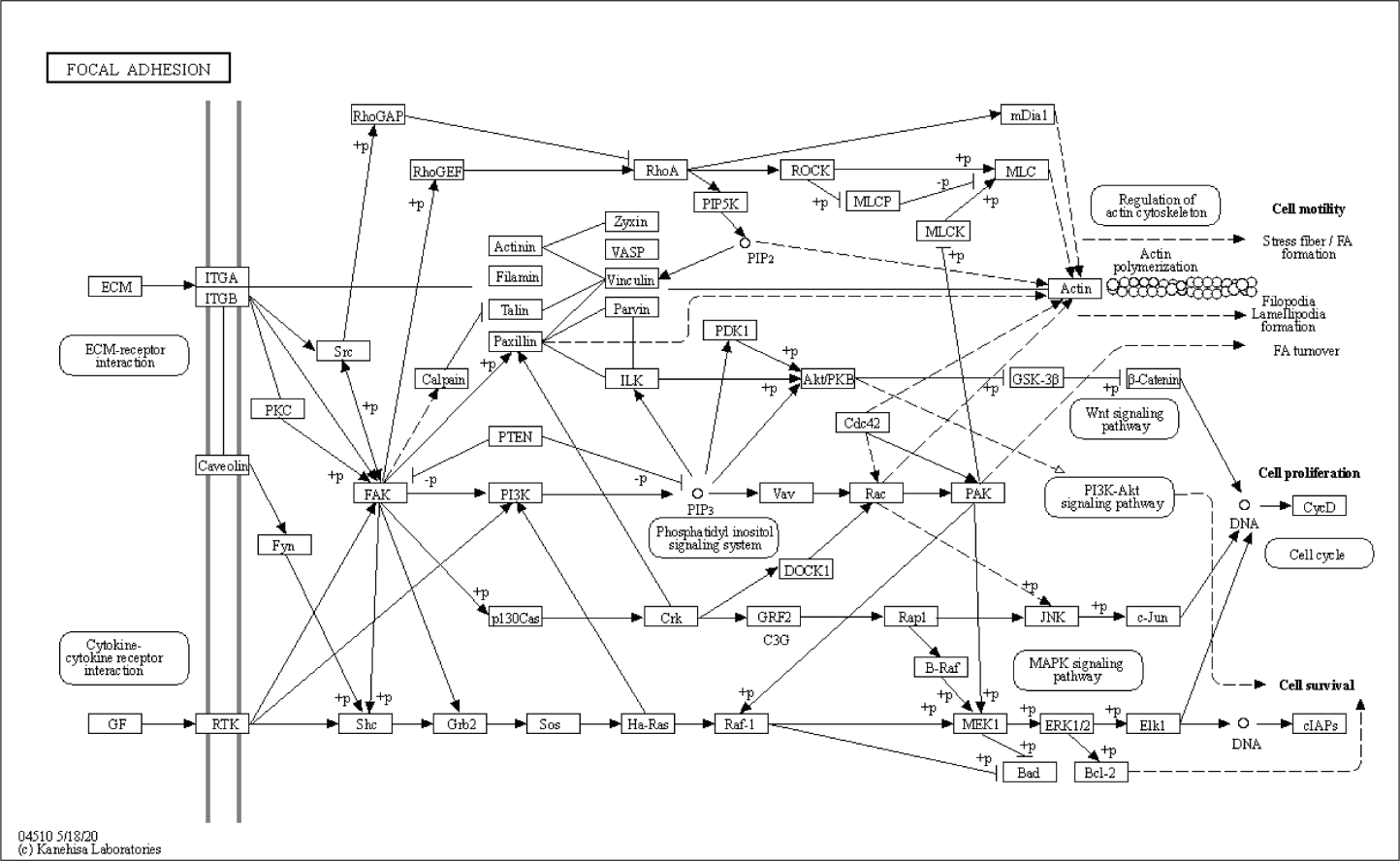
**

**Supplementary Figure 3. Focal adhesion (hsa04510) pathway in KEGG database**
